# Supplementary material for: Human DNA replication initiation sites are specified epigenetically by oxidation of 5-methyl-deoxycytidine
Source: Nucleic Acids Res. 2025 May 5;53(8):gkaf362. doi: 10.1093/nar/gkaf362 (PMC12051107; doi:10.1093/nar/gkaf362)
Supplement: gkaf362_Supplemental_File [file gkaf362_supplemental_file.pdf]

Supplementary information for:

Human DNA replication initiation sites are specified epigenetically by oxidation of 5-methyl-deoxycytidine

Torsten Krude[1*], Jiaming Bi[1], Rachel Doran[1], Rebecca A. Jones[2,3], James C Smith[2]

[1] Department of Zoology, University of Cambridge, Downing Street, Cambridge, CB2 3EJ, UK

[2] Developmental Biology Laboratory, Francis Crick Institute, 1 Midland Road, London NW1 1AT, UK

[3] Present address: Department of Molecular Biology, Princeton University, Princeton, USA

* Corresponding author: tk218@cam.ac.uk

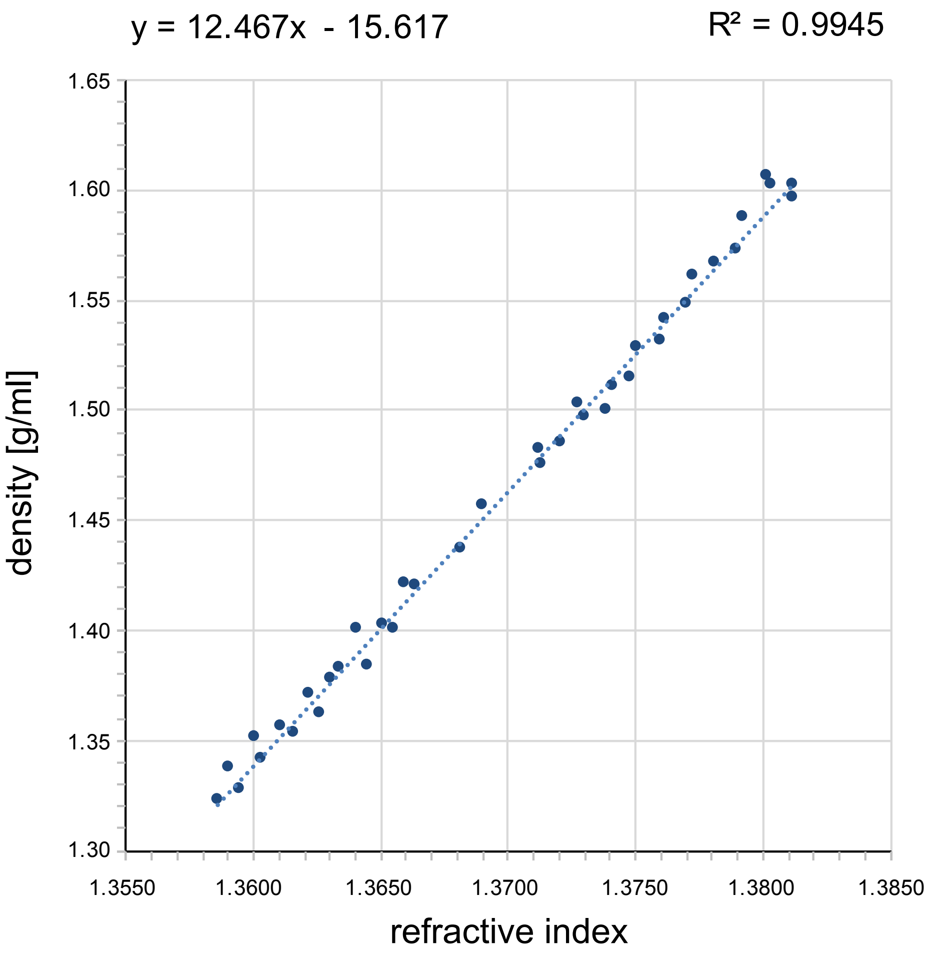

Figure S1

Conversion of refractive index to density

Plot of experimentally determined densities (D) and refractive indexes (RI) of n=40 fractions across two caesium sulphate density equilibrium gradients. The linear regression follows the equation: D = 12.46 x RI x g/ml -15.62 (R^2 = 0.9945).

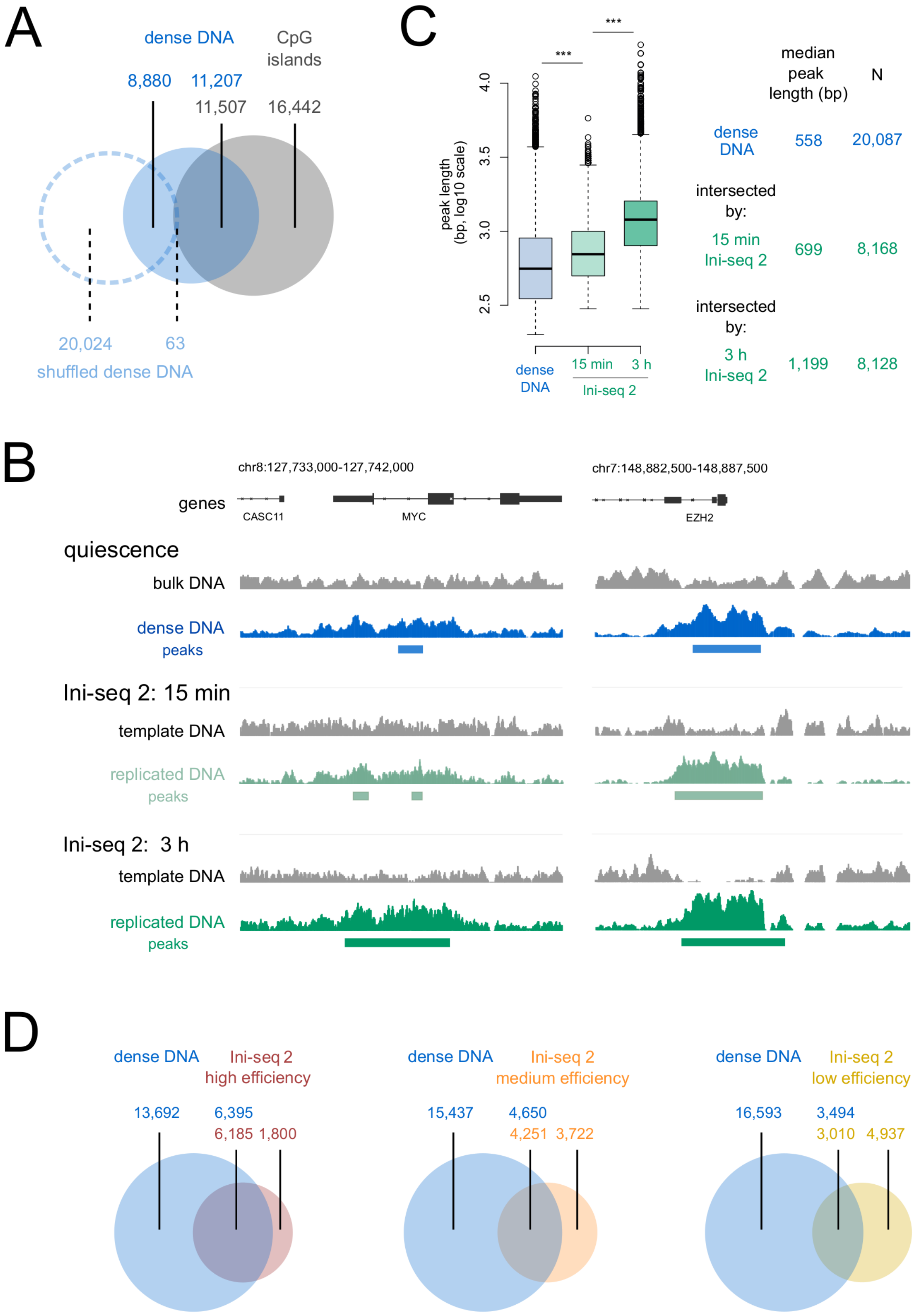
A
dense DNA
CpG islands
8,880
11,207
11,507
16,442
20,024
63
shuffled dense DNA
C
***
***
peak length (bp, log10 scale)
2.5
3.0
3.5
4.0
dense DNA
15 min
3 h
Ini-seq 2
median peak length (bp)
N
dense DNA
558
20,087
intersected by:
15 min Ini-seq 2
699
8,168
intersected by:
3 h Ini-seq 2
1,199
8,128
B
chr8:127,733,000-127,742,000
chr7:148,882,500-148,887,500
genes
CASC11
MYC
EZH2
quiescence
bulk DNA
dense DNA
peaks
Ini-seq 2: 15 min
template DNA
replicated DNA
peaks
Ini-seq 2: 3 h
template DNA
replicated DNA
peaks
D
dense DNA
Ini-seq 2 high efficiency
13,692
6,395
6,185
1,800
dense DNA
Ini-seq 2 medium efficiency
15,437
4,650
4,251
3,722
dense DNA
Ini-seq 2 low efficiency
16,593
3,494
3,010
4,937

Figure S2

Naturally dense DNA is enriched at active DNA replication origins

(A) Genome-wide intersect analyses between MACS2 peaks of dense DNA and CpG islands. Non-intersected and intersected numbers of peaks are indicated; the top number in the intersect indicates the number of peaks of the left distribution (dense DNA) intersecting with the right distribution (CpG islands), and the bottom number indicates the number of peaks of the right intersecting with the left distribution. An intersect analysis using shuffled dense sites after randomisation per chromosome are included as hatched circles, together with corresponding peak numbers (in pale blue). **(B-D)** Integration of dense DNA with original ini-seq 2 replication origin mapping data (Guilbaud et al., 2022). **(B)** Illumina sequencing read coverage profiles of bulk and dense DNA from quiescent cells (grey and blue) are compared to ini-seq 2 profiles of unreplicated template DNA (grey) and replicated DNA (green) at the low efficiency MYC (left) and the high efficiency EZH2 origin sites (right) after 15 min and 3 h incubations. Genome coordinates and positions of reference genes are indicated. Enrichment peaks were called by MACS2 for the dense DNA, and by a custom algorithm based on conversion of unreplicated template DNA to replicated DNA (Guilbaud et al., 2022). Peak positions are indicated by solid bars underneath the read coverage profiles. Note the increasing size of replicated DNA peaks and the local reduction of unreplicated template DNA and concomitant accumulation of replicated DNA over replication time at origin sites marked in quiescence by dense DNA. **(C)** Distributions of peak lengths for dense DNA in quiescent cells (blue), of ini-seq 2 origins detected after 15 min incubation time that intersect with these dense peaks (pale green), and of ini-seq 2 origins detected after 3 h incubation time that intersect also with those dense ini-seq 2 15-min origin sites (green). Peak lengths are plotted on a logarithmic scale to the base of 10, and statistical significance is indicated between distributions (Kolmogorov-Smirnov tests: ***, $p < 2e-16$). Tabulated median and N values for these distributions are included on the right. **(D)** Intersect analyses of MACS2 peaks of dense DNA with high-, medium- and low-efficiency class ini-seq 2 origin peaks (Guilbaud et al., 2022).

A

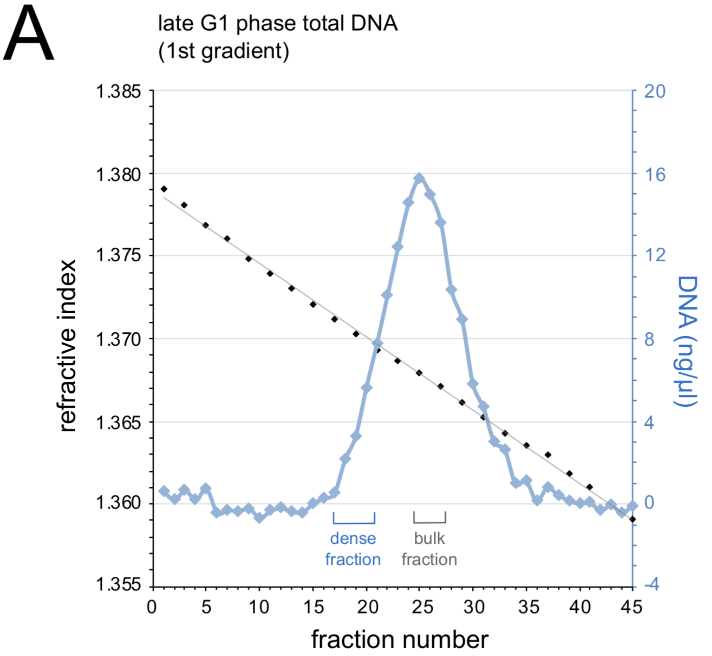

B

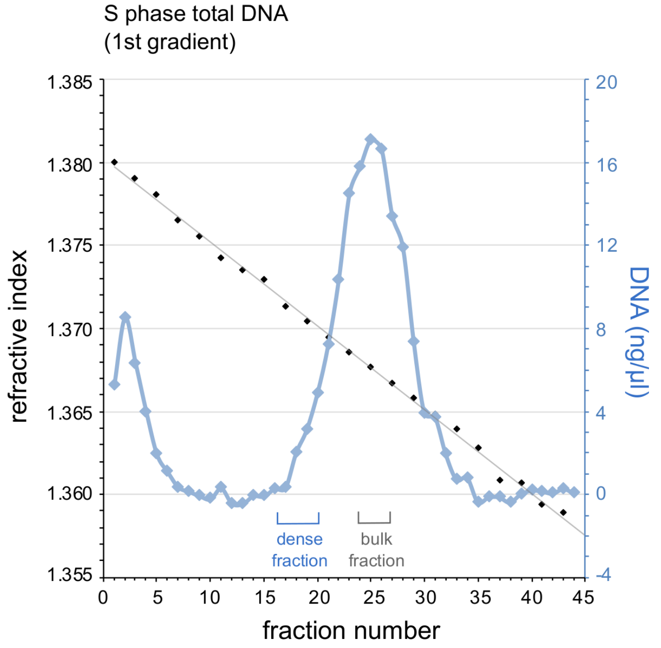

Figure S3

Density gradient separation of fragmented late G1 and S phase human DNA

Human EJ30 cells were synchronised in late G1 phase by mimosine (Krude, 1999) and in S phase by thymidine (Krude et al., 1997). Genomic DNA was purified, fragmented and separated on primary caesium sulphate density equilibrium gradients. **(A)** Separation of late G1 phase cell DNA. Isolated dense DNA fractions for loading onto a second gradient and isolated bulk DNA fractions selected for sequencing and PCR analysis are indicated. **(B)** Separation of S phase cell DNA. See Figure 3 for secondary density gradients and further analysis.

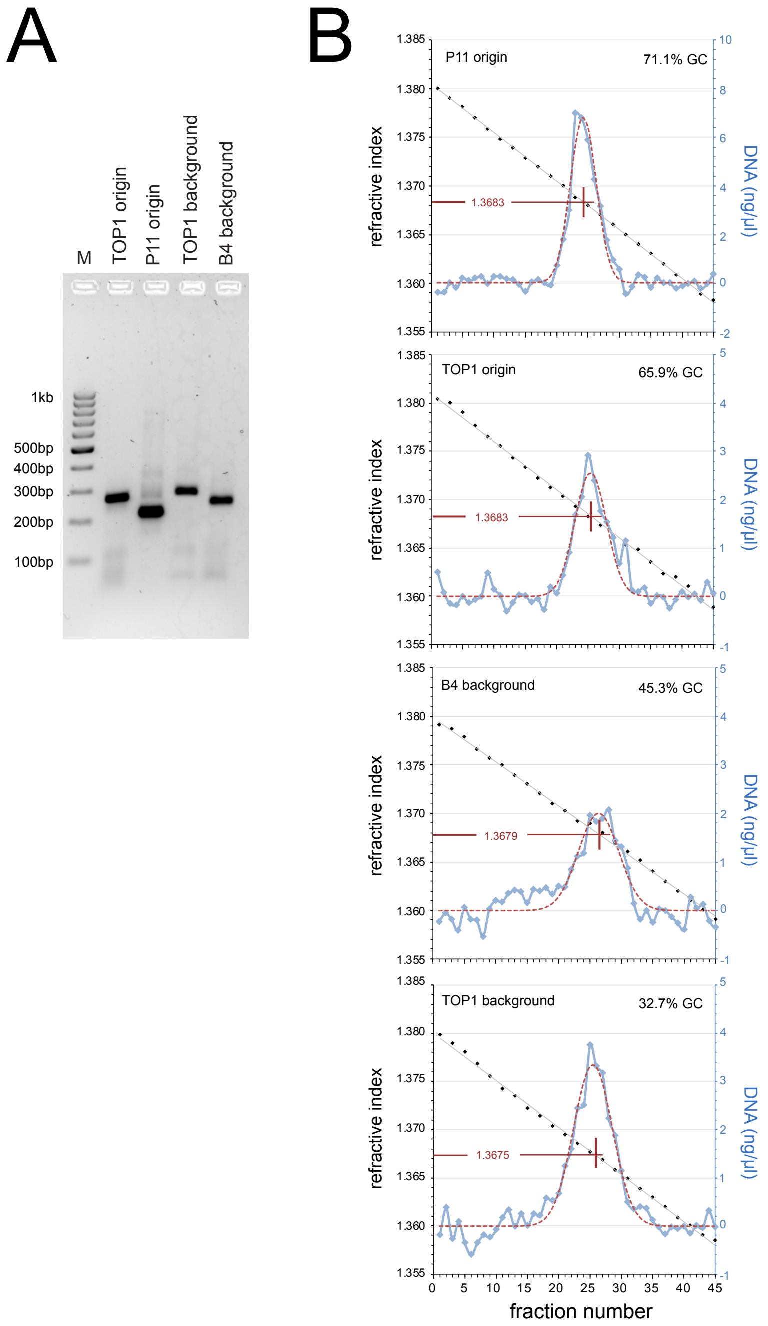

Figure S4

Dense DNA is a consequence of oxidised methyl-deoxycytidines

(A, B) Analysis of unmodified DNA fragments. **(A)** Synthesis of defined DNA fragments. PCR products of two selected origin (TOP1 and P11) and corresponding background sites (TOP1 and B4) are separated by agarose gel electrophoresis. M, 100 bp ladder. See Table S1 below for DNA sequences of these fragments. **(B)** Comparative density analysis of these defined DNA fragments with different GC content. PCR products were synthesised using unmodified dCTP and separated on density gradients. DNA fragment identities with their %GC content are indicated for each gradient, together with fitted Gauss distributions (hatched red lines), and the refractive indexes for the means of each fitted distribution.

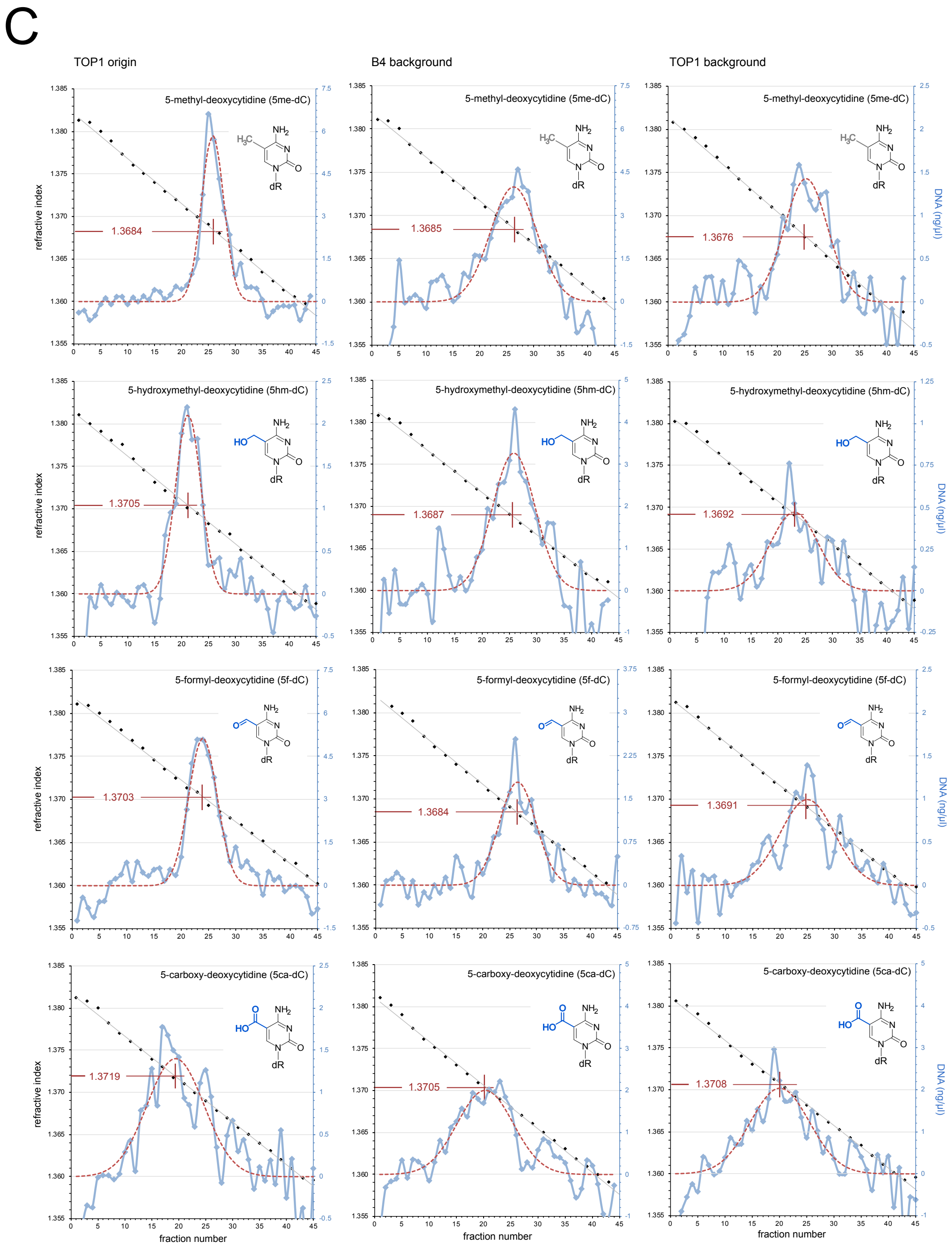

Figure S4, continued

Dense DNA is a consequence of oxidised methyl-deoxycytidines

(C) Comparative density analysis of methylated and further oxidised DNA fragments of the TOP1 origin and B4 and TOP1 background sites (left to right columns). PCR products were synthesised using modified dCTP for an incorporation of 5me-dC, 5hm-dC, 5f-dC, and 5ca-dC (top to bottom rows). DNA fragment identities, deoxycytidine modifications, DNA concentrations (pale blue), together with fitted Gauss distributions (hatched red lines), and the refractive indexes for the means of each fitted distribution are indicated. Note the increased densities for DNA fragments containing 5hm-dC, 5f-dC, and 5ca-dC.

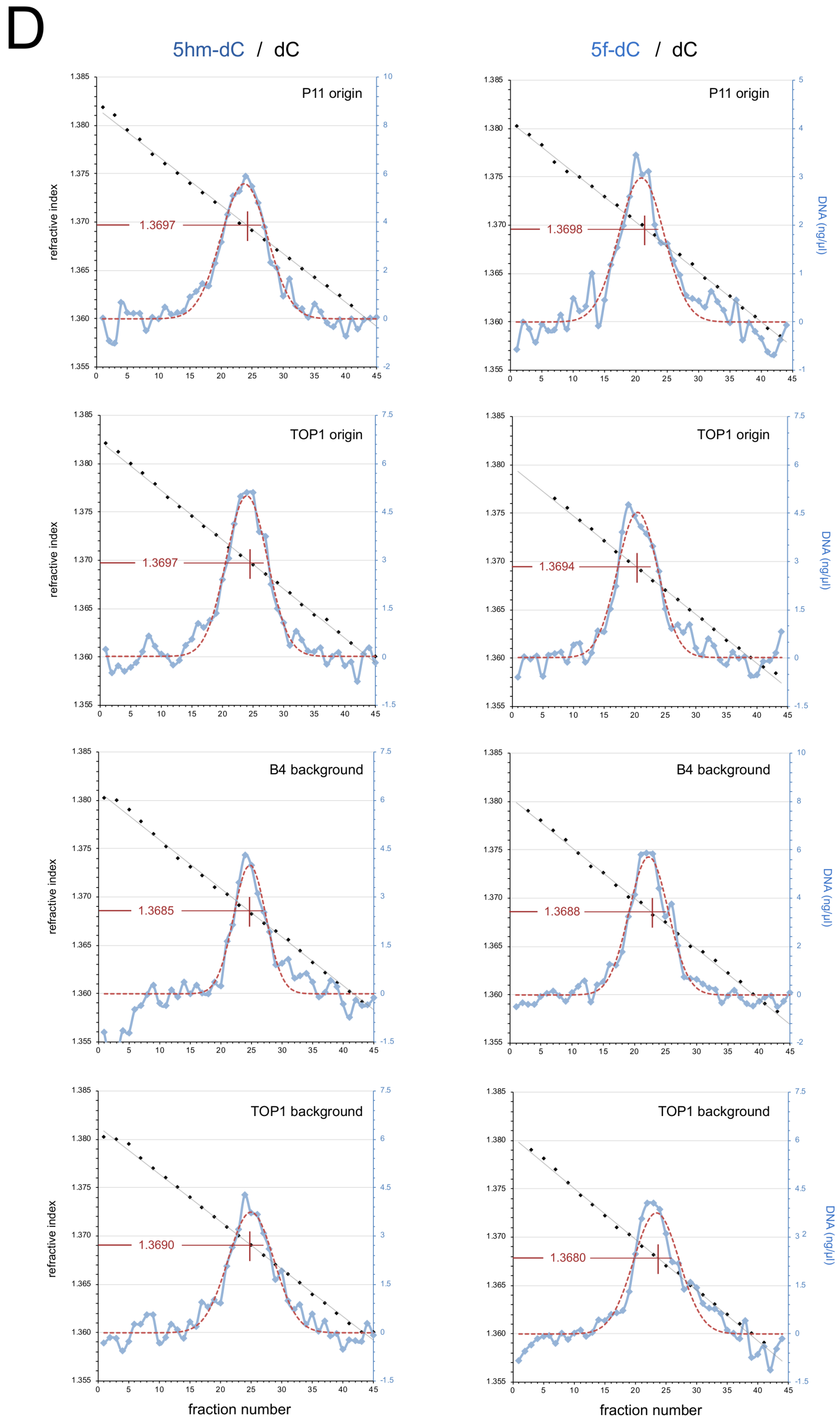

Figure S4, continued

Dense DNA is a consequence of oxidised methyl-deoxycytidines

(D) Comparative density analysis of hemi-modified DNA fragments. Hemi-modified double stranded DNA containing either 5hm-dC (left column) or 5f-dC (right column) in only one DNA strand was synthesised for the indicated DNA fragments by PCR, using long unmodified primers spanning about half the length of the DNA fragment. The polarity of the modified strand therefore changes midway at the converging 3' ends of the unmodified primers. Density gradient profiles are shown as defined for panel C.

A

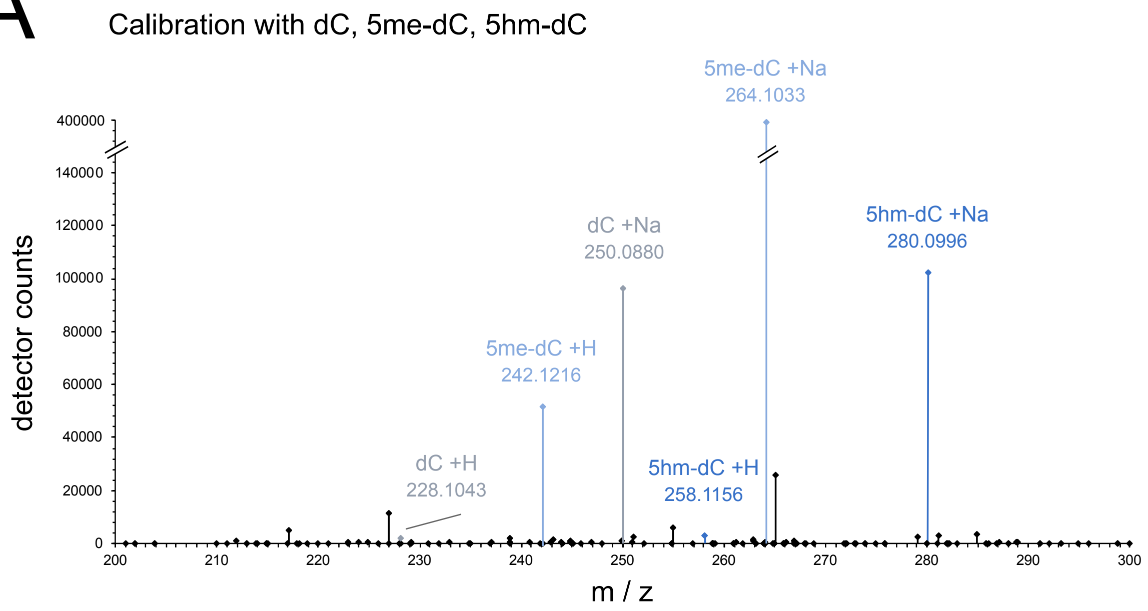

B

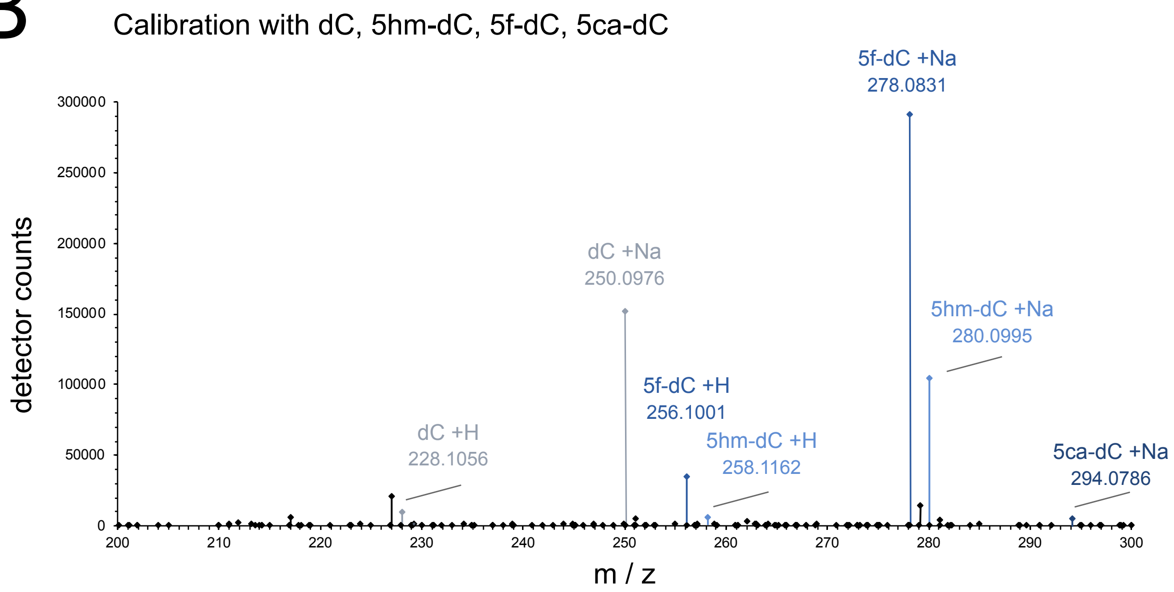

Figure S5

Calibration of mass spectra using defined deoxycytidine derivatives

Two separate combinations of nucleoside triphosphates were dephosphorylated and subjected to LS-MS: **(A)** dCTP, 5me-dCTP, 5hm-dCTP. **(B)** dCTP, 5hm-dCTP, 5f-dCTP and 5ca-dCTP. Plots of detector counts across the mass-over-charge (m/z) range of 200-300 Da are shown. Peaks corresponding to the monoisotopic masses of the indicated nucleosides including H and Na adducts are highlighted.

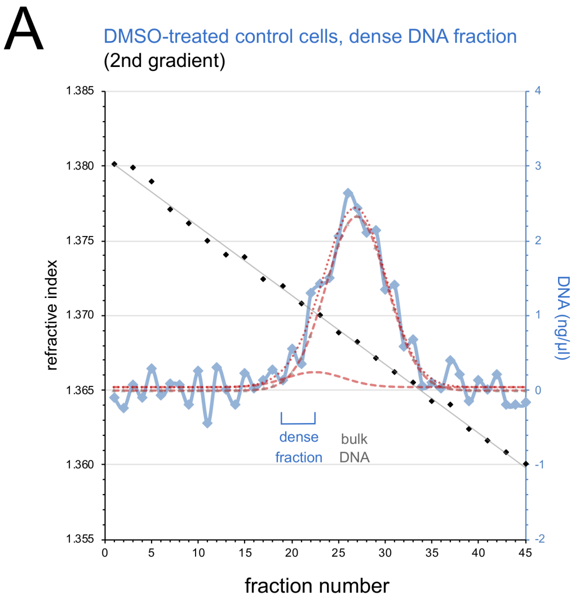

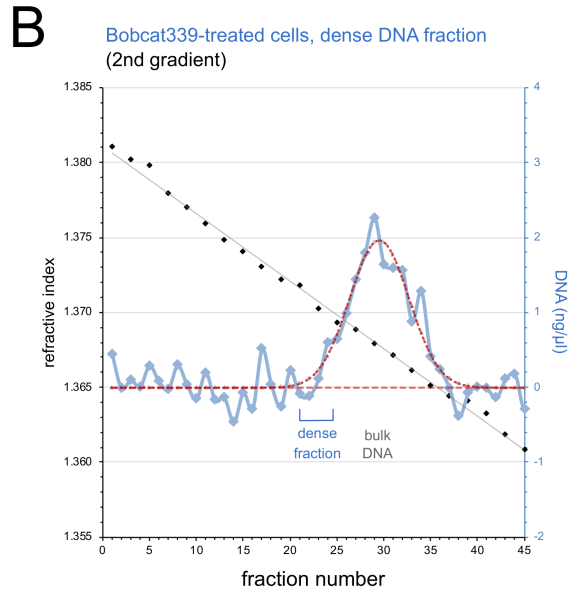

Figure S6

Methylation of deoxycytidine and its further oxidation is required for cell proliferation and DNA replication

(A, B) Density gradient analysis of fragmented DNA from DMSO and Bobcat339-treated cells. **(A)** Analysis of dense DNA from asynchronously proliferating, DMSO-treated EJ30 cells. **(B)** Analysis of dense DNA from EJ30 cells treated with 125 µM of Bobcat339. Dense fractions from a primary density gradient were taken and run on a second caesium sulphate density gradient. For each gradient, calculated best fits to two normal Gaussian distributions are plotted by hashed (individual) and dotted (combined) red lines for dense and bulk DNA. Positions of reference bulk DNA, and of isolated dense DNA fractions selected for further analysis (shown in Fig. 6B) are indicated.

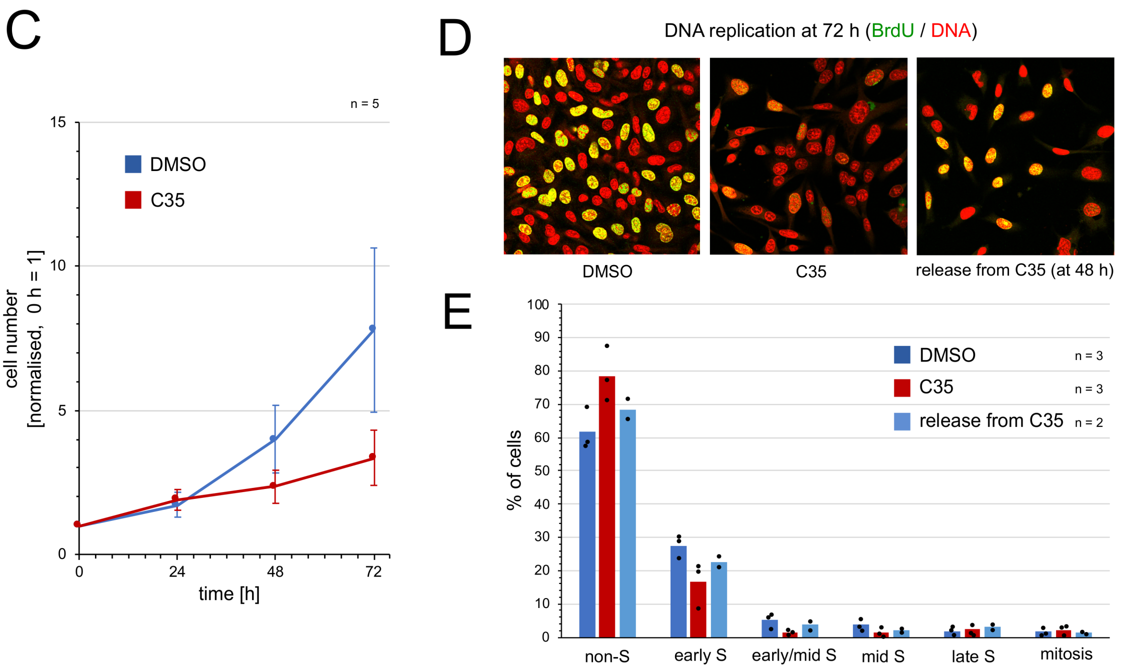

Figure S6, continued

Methylation of deoxycytidine and its further oxidation is required for cell proliferation and DNA replication

(C-E) Inhibition of TET enzyme activity by TET inhibitor C35. **(C)** Inhibition of cell proliferation. Asynchronously proliferating EJ30 cells were treated with DMSO (blue) or 150 µM of C35 (red) for the indicated times. Normalised cell numbers are plotted against time as averages ± standard errors of the mean for n independent experiments. **(D, E)** Inhibition of DNA replication. After 72 h of treatments, including a release of C35-treated cells into fresh medium at 48 hours, replicating cells were pulse-labelled with BrdU and visualised by confocal immunofluorescence microscopy. **(D)** Representative micrographs. DNA is stained with propidium iodide (red), BrdU incorporation is detected with specific antibodies (green). Note the lower proportions of replicating cell nuclei and lower intensity of BrdU incorporation signals in C35-treated cells compared to the DMSO control and the release. **(E)** Quantification of the percentages of cells in the indicated stages of the cell cycle. Mean values are plotted as histogram bars and individual data points are superimposed for n independent experiments.

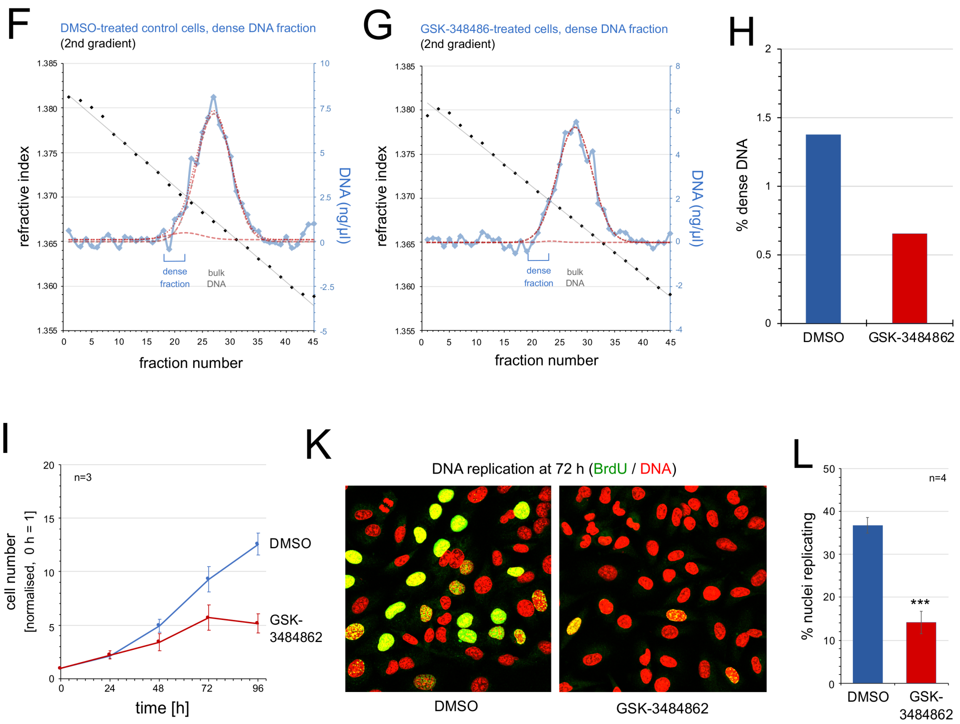

Figure S6, continued

Methylation of deoxycytidine and its further oxidation is required for cell proliferation and DNA replication

(F-L) Inhibition of DNMT activity by GSK-3484862. **(F)** Separation of dense DNA from asynchronously proliferating, DMSO-treated cells on a second caesium sulphate density gradient. Calculated best fits to two normal Gaussian distributions are plotted by hashed (individual) and dotted (combined) red lines for dense and bulk DNA, and isolated dense DNA fractions selected for further analysis are indicated. **(G)** Separation of dense DNA from cells treated with 10 µM of GSK-3484862 on a second caesium sulphate density gradient. **(H)** Quantification of relative dense DNA amounts. Relative amounts were obtained as percentages of dense DNA out of total DNA from the DMSO and GSK-3484862-treated cells. **(I)** Inhibition of cell proliferation. Asynchronously proliferating EJ30 cells were treated with DMSO (blue) or 10 µM of GSK-3484862 (red) for 96 h. Normalised cell numbers are plotted against time as averages ± standard errors of the mean for n=3 independent experiments. **(K, L)** Inhibition of DNA replication. After 72 h of treatment, replicating cells were pulse-labelled with BrdU and replicating cell nuclei detected by confocal immunofluorescence microscopy. **(K)** Representative micrographs. Note the lower proportions of replicating cell nuclei and lower intensity of BrdU incorporation signal in GSK-3484862-treated cells compared to the DMSO control. **(L)** Quantification of the percentages of replicating cell nuclei. Proportions of nuclei replicating their DNA were scored and plotted. Mean values ± standard errors of the mean from n=4 independent experiments are shown (T-tests, two-tailed, unequal variance between DMSO control and treatment: ***, p<0.001).

Table S1

DNA sequences of amplified PCR products (5' – 3').
Additional Adenosine residues added to the 3' end of each strand during PCR by Taq DNA polymerases are shown in grey. MspI restriction sites are indicated in bold.

TOP1 ori (TOP1 origin site)

TCCTTATGCAAATCACAGCGGAGCGCGCACGGT**CCGG**AGGCGGGGCTTGCGATGCAAAGACA
GGTCCGTCTGGCGAACAGCGAGGGGGCGGGCCGCAACCCTCTGCCTCTTTCCGCGAGCGCTG
ACGTCGCCGACGTGTTGTTTAAAAGCGGCCGCGCAGGCGCAGTGAGCCCAAATGCGAACTTA
GGCTGTTACACAACTGCTGGGGTCTGTTCTCGCCGCCCGC**CCGG**CAGTCAGGCAGCGTCGCC
GCCGTGGTAGCAGCCTA

(265bp, 64%GC)

TOP1 bg (TOP1 background site)

TCAAAATTGGGCTGTGAGGTTTTTTTGTTTTGCTTGTTTTCAGTGCATGTGTGGGGTGGGGA
GGTGCAAAAAAATGTTTGCCTTCTAATATACAGCTTTGTTGTATATTAATTATATAGTAACA
GTTGTCCCTGCTGCTAATGGTATGGAAAATGATTCTGTTGTGCTTTCTGAATACATATCATT
TTAGAAGTTTCAGATTATACTAAAACCATGTTATTTCATATTAGTTGGACATGAATGCATAT
TTATATGACACATCATTATCAACTTTTGCCTA

(280bp, 31%GC)

P11 ori (PDIA4 origin site)

TAGCTGCACCAGCCCCAAGAGCAGCAGGAGCAGGAAGGCTTT**CCGG**GGCCTCATGGTAGCGG
GGGCGGAGCGCGGCCTCCTAGCGTCGGCGGCCGCTGAGCGCACCGAGAACTCGGGGTCTGGC
CGACAGCCCGTCGCTCCTTAGCGACGCGGGGGAG**CCGG**AAAAACCCACGGAAGTCGTCCCCG
GCGATTGGCAGGGGGCGGAGGAAGTCGCGGG**CCGG**CCAATCCCAGACTGACGCCGA

(242bp, 70%GC)

B4 bg (PDIA4 background site)

TACCAGTTTCAGGATAAGGCTGTTTCTTTTTTTTTAGATATTTGCTGAGGGCTGAAACAAGCA
AGCTGTTAATGCCTGAACTTTTTTCCTACCAAGTCCCTAGTGCTTCATATTAGTATGTACAT
GGTCAGGTCCCAAGAAACAGCAGGTGATAGCTCCCACCCAACAAAGACTGTCAACTTCCTGA
ACCAGGACAGTCCTCACTTCCCTCCATCCCACTCTTTGCGGCCAATTCCATAGTTTAGGAA

(247bp, 43%GC)

Table S2

DNA sequences of long PCR primers.

Primers are used for the synthesis of hemi-modified DNA fragments. Primer sequences are shown in 5' – 3' direction.

TOP1 ori (origin site) F
CCTTATGCAAATCACAGCGGAGCGCGCACGGTCCGGAGGCGGGGCTTGCGATGCAAAGACAG
GTCCGTCTGGCGAACAGCGAGGGGGCGGGCCGCAACCCTCTGCCTCTT

TOP1 ori (origin site) R
AGGCTGCTACCACGGCGGCGACGCTGCCTGACTGCCGGGCGGGCGGCGAGAACAGACCCCAG
CAGTTGTGTAACAGCCTAAGTTCGCATTTGGGCTCACTGCGCCTGCGCGGCCGCTT

TOP1 bg (background site) F
CAAAATTGGGCTGTGAGGTTTTTTTGTTTTGCTTGTTTTCAGTGCATGTGTGGGGTGGGGAG
GTGCAAAAAAATGTTTGCCTTCTAATATACAGCTTTGTTGTATATTAATTATA

TOP1 bg (background site) R
AGGCAAAAGTTGATAATGATGTGTCATATAAATATGCATTCATGTCCAACTAATATGAAATA
ACATGGTTTTAGTATAATCTGAAACTTCTAAAATGATATGTATTCAGAAAGCA

P11 ori (PDIA4 origin site) F
AGCTGCACCAGCCCCAAGAGCAGCAGGAGCAGGAAGGCTTTCCGGGGCCTCATGGTAGCGGG
GGCGGAGCGCGGCCTCCTAGCGTCGGCGGCCGCTGAGCGCACCGAGAACTCGGGGTCT

P11 ori (PDIA4 origin site) R
CGGCGTCAGTCTGGGATTGGCCGGCCCGCGACTTCCTCCGCCCCCTGCCAATCGCCGGGGAC
GACTTCCGTGGGTTTTTCCGGCTCCCCCGCGTCGCTAAGGAGCGACGGGCTGTC

B4 bg (PDIA4 background site) F
ACCAGTTTCAGGATAAGGCTGTTTCTTTTTTTTAGATATTTGCTGAGGGCTGAAACAAGCAA
GCTGTTAATGCCTGAACTTTTTTCCTACCAAGTCCCTAGTGCTTCATATTAGTATGTA

B4 bg (PDIA4 background site) R
TCCTAAACTATGGAATTGGCCGCAAAGAGTGGGATGGAGGGAAGTGAGGACTGTCCTGGTTC
AGGAAGTTGACAGTCTTTGTTGGGTGGGAGCTATCACCTGCTGTTTCTTGGGACCTGA

Supplementary references

Guilbaud, G., Murat, P., Wilkes, H.S., Lerner, L.K., Sale, J.E., Krude, T., 2022. Determination of human DNA replication origin position and efficiency reveals principles of initiation zone organisation. Nucleic Acids Res 50, 7436–7450.

Krude, T., 1999. Mimosine Arrests Proliferating Human Cells before Onset of DNA Replication in a Dose-Dependent Manner. Experimental Cell Research 247, 148–159.

Krude, T., Jackman, M., Pines, J., Laskey, R.A., 1997. Cyclin/Cdk-Dependent Initiation of DNA Replication in a Human Cell-Free System. Cell 88, 109–119.
